# Supplementary material for: Hsa_circ_0006692 Promotes Lung Cancer Progression via miR-205-5p/CDK19 Axis
Source: Genes (Basel). 2022 May 10;13(5):846. doi: 10.3390/genes13050846 (PMC9141027; doi:10.3390/genes13050846)
Supplement: Supplementary file 1 [file genes-13-00846-s001.zip › genes-1701796-supplementary.pdf]

Supplementary Table1. The list and sequence of long primers used for RT-qPCR analysis

Gene	Sequence（5’-3’）	Length(bases)
circ-0006692		
F-primer	CGGATGCGTGTGAGAAAGT	20
R-primer	CTCGCTGTCACAGTTGGGTA	20
CDK19		
F-primer	ATGCAGAGCATGACTTGTGG	20
R-primer	GACTCTCCCCCTCTCAGGAC	20
MMP7		
F-primer	TGGGGAACTGCTGACATCAT	20
R-primer	CCCTAGACTGCTACCATCCG	20
CDH1		
F-primer	CGGACGATGATGTGAACACC	20
R-primer	TTGCTGTTGTGCTTAACCCC	20
CDH2		
F-primer	CGGTTTCATTTGAGGGCACA	20
R-primer	TTGGAGCCTGAGACACGATT	20
VIM		
F-primer	GAGTCCACTGAGTACCGGAG	20
R-primer	ACGAGCCATTTCCTCCTTCA	20
PCNA		
F-primer	GGCGTGAACCTCACCAGTAT	20
R-primer	TCTCGGCATATACGTGCAAA	20
CCND1		
F-primer	CTGCGAAGTGGAAACCATC	20
R-primer	CACATCTCCAGCATCCAGGT	20
BCL2		
F-primer	GCCTTCTTTGAGTTCGGTGG	20
R-primer	GAAATCAAACAGAGGCCGCA	20
